# Analysis of electric moments of RNA-binding proteins: implications for mechanism and prediction

**DOI:** 10.1186/1472-6807-11-8

**Published:** 2011-02-01

**Authors:** Shandar Ahmad, Akinori Sarai

**Affiliations:** 1 National Institute of Biomedical Innovation, 7-6-8, Saito-asagi, Ibaraki, Osaka, Japan; 2 Department of Bioscience and Bioinformatics, Kyushu Institute of Technology, Iizuka, Fukuoka, 820-8502 Japan

## Abstract

**Background:**

Protein-RNA interactions play important role in many biological processes such as gene regulation, replication, protein synthesis and virus assembly. Although many structures of various types of protein-RNA complexes have been determined, the mechanism of protein-RNA recognition remains elusive. We have earlier shown that the simplest electrostatic properties viz. charge, dipole and quadrupole moments, calculated from backbone atomic coordinates of proteins are biased relative to other proteins, and these quantities can be used to identify DNA-binding proteins. Closely related, RNA-binding proteins are investigated in this study. In particular, discrimination between various types of RNA-binding proteins, evolutionary conservation of these bulk electrostatic features and effect of conformational changes by complex formation are investigated. Basic binding mechanism of a putative RNA-binding protein (HI1333 from Haemophilus influenza) is suggested as a potential application of this study.

**Results:**

We found that similar to DNA-binding proteins (DBPs), RNA-binding proteins (RBPs) also show significantly higher values of electric moments. However, higher moments in RBPs are found to strongly depend on their functional class: proteins binding to ribosomal RNA (rRNA) constitute the only class with all three of the properties (charge, dipole and quadrupole moments) being higher than control proteins. Neural networks were trained using leave-one-out cross-validation to predict RBPs from control data as well as pair-wise classification capacity between proteins binding to various RNA types. RBPs and control proteins reached up to 78% accuracy measured by the area under the ROC curve. Proteins binding to rRNA are found to be best distinguished (AUC = 79%). Changes in dipole and quadrupole moments between unbound and bound structures were small and these properties are found to be robust under complex formation.

**Conclusions:**

Bulk electric moments of proteins considered here provide insights into target recognition by RNA-binding proteins, as well as ability to recognize one type of RBP from others. These results help in understanding the mechanism of protein-RNA recognition, and identifying RNA-binding proteins.

## Background

Protein-RNA interactions have been identified as crucial for a number of cellular processes [1-7]. However, the mechanism of RNA recognition by proteins or vice versa has been poorly understood despite a recent surge in the study of protein-RNA interactions for specific systems as well as their statistical analysis and prediction [8-12]. Most computational studies on protein-RNA interactions have focused on classification, annotation and binding-site characterization [11,12]. A large number of features have often been employed for accurate predictions of these RNA-binding proteins as well as their interface residues [12]. Structure-based predictions and analysis of RBP's have focused on high-resolution structures utilizing detailed structural parameters such as solvent accessibility and detailed geometrical features such as cleft and patch. In these studies, basic electrostatic features such as dipole and quadrupole moments are typically considered in combination with many other parameters (e.g., 40 parameters by Shazman and Gutfreund [12]), which prevents us from looking at the role of individual physical properties of proteins in RNA-recognition and therefore some of the obvious role of electrostatic interactions may be lost in an effort to maximize prediction performance. We have earlier shown that simple electrostatic properties viz. net charge, dipole and quadrupole moments carry significant information useful to predict DNA-binding proteins both from full atomic coordinates as well as main chain atoms [13]. Subsequent studies confirmed that low-resolution structures could be used to apply this method to the prediction of nucleic acid binding function [14]. A Web-based tool to calculate dipole and quadrupole moments and reflections on their relationship to functional protein classes has also become available and was published recently [15].

Here, we carry out a systematic analysis of three bulk electrostatic properties of RNA-binding proteins (RBPs) viz. net charge, dipole and quadrupole moments, all calculated from low-resolution protein structures with only main-chain coordinates, in order to estimate how far these simple properties are able to identify RBPs from control proteins. Simple statistical analysis of electric moments in each category has been supplemented by all-against-all pair-wise recognition of various types, determined by neural network prediction. Our results show that there exists a pattern of electric moments in RBPs, which is different from the control data as well as within the proteins binding to various types of RNAs. One type of RNA-binding proteins can be distinguished from the other on the basis of these properties with various degrees of accuracy. Finally, we compile a data set of pairs of structures of the same RBPs solved in monomer state as well as full protein-RNA complex. Using this data set, we show that the calculation of moments is rather robust against conformational changes induced by complex formation. Finally, we discuss possible implications of the present results for the mechanism of protein-RNA interactions.

## Methods

### Data set of RNA-binding proteins

Primary source of RNA-binding proteins and their annotations into various categories is SCOR database [16]. First, a list of all PDB codes present in SCOR was compiled, resulting in 569 entries. All 569 PDB entries were scanned for RNA (998 chains) and proteins (1435 chains). Protein chains were then scanned to be in direct contact with at least one RNA chain. Proteins with at least 3 residues in contact were selected, resulting in 1242 chains. FASTA-formatted protein sequences were generated from the PDB files and redundancy was removed by clustering them at 25% sequence identity using BLASTCLUST [17]. This resulted in RBP_NR25 database of 160 protein chains, to be subsequently referred to as simply RBP. SCOR functional classification was used to annotate them as binding to mRNA (13 chains), tRNA (20 chains), rRNA (84 chains) or viral RNA (17 chains). Final list of selected protein chains, their calculated moments, along with other data sets, is provided in Additional File 1.

### Development of control data sets

First, a non-redundant list of all protein chains in PDB was obtained from PDBselect [18]. The latest (May30-2010 version) PDBselect (25% sequence ID clusters) consisted of 4868 protein chains. From this, chains smaller than 50 residues were removed, which resulted in 4133 protein chains. Next, a keyword search using "Nucleic acid binding" was carried out in SWISSPROT and resulting 20595 proteins chains were obtained in this way. Then, the 4133 chains selected from PDBselect were aligned against all the 20595 SWISSPROT sequences to obtain any similarity, using BLAST at e-values cutoff of 0.01. These chains were excluded from PDB select sequence database. Further PDB entry type was checked and nucleic acid binding chains were removed, leaving 2441 sequences with no similarity to RNA binding proteins with known or unknown structure were obtained. These 2441 protein chains were used as a control data set for all our analysis (see Additional File 1).

### Complex versus monomeric structure pairs

Sequence homologues of proteins used in the above data set (RBP_NR25) were searched in PDB with at least 90% sequence identity and the best match was selected. Minimum alignment coverage was also set at 90% and only those target sequences that occurred in monomeric PDB entries were selected.

### Calculations of electric moments

Charge, dipole moment and quadrupole moments were calculated as described in our earlier study [13]. According to that study, consideration of all-atom coordinates did not affect the overall results, as compared to the low-resolution model with only backbone coordinates. Thus, in this study, side-chain coordinates of the proteins were ignored and the electric moments were based on the main chain conformation determined by C_α_-position of the residues. All Lys and Arg residues were assigned a positive charge and Glu and Asp residues were considered negative. All other residues were treated as neutral: His was considered as neutral, as the consideration of its charged states had negligible effects (see Results section). All water molecules, metals and ligands were also ignored for these calculations.

Components of dipole moments were calculated using the expression

(1) $$P=\Sigma \left(R_{i}-R_{o}\right)\cdot q_{i}$$

where R_o _is the reference point, which was taken as the geometric center of all the residues (C_α_-positions) in the structure, and i represents an atom in the protein structure. Net dipole moment was calculated by taking a vector sum of these components.

Quardupole moment is a tensor of rank 2 and a direct calculation from the PDB coordinates gives nine components (*M*_xx_, *M*_xy_, *M*_xz_, *M*_yx_, *M*_yy_, *M*_yz_, *M*_zx_, *M*_zy _and *M*_zz_). Each of these components is calculated by the following expression

(2) $$M_{\alpha \beta }=1/2\;\Sigma \left(3\;r_{\text{i}\alpha }r_{\text{i}\beta }-r_{\text{i}}{}^{2}\delta_{\alpha \beta }\right)\;\cdot \;q_{i}$$

where *r*_i _is the relative position vector, i is the index of charge and summation is over all charges. The quadrupole moment matrix can be diagonalized and the three eigenvalues of the quadrupole moment matrix are represented as Q1, Q2 and Q3 in decreasing order. We used the largest eigenvalue Q1 for designating single quadrupole moment and all three eigen values for developing the predictor.

All electric moment values were the absolute values and normalized by the protein sequence length in a way similar to our earlier study [13]. Units are often omitted in describing quadrupole moments and net charge as these values are measured in atomic units (using electronic charge and Å as charge and distance units in calculations). Dipole moment values are quoted by converting them to Debyes.

Our method of computing electric moments is somewhat different from a similar approach adopted in a recently published dipole moment server [15]. First of all, we use only the C_α _atoms for assigning charges, whereas charges are assigned to specific atomic positions in [15]. Secondly, we used geometric center of all C_α _atoms (including residues with zero charge assignments to compute the reference point and axes) and finally, we obtain quadrupole moments by taking their eigen values, which is not provided in [15]. We find that there is a moderate correlation (~0.5) between the dipole moments computed by the two methods. Since our approach is more suitable for low resolution structures (does not require side chain positions), we report only the results obtained by our procedure. For similar reasons, we did not try to predict protonation state of residues, which could sometimes be possible if side-chain coordinates are provided [19].

### Statistical significance of difference

Distributions of moments between control and RNA-binding as well as between various classes of RNA-binding proteins were compared by measuring the statistical significance of difference between their means. A two-tailed Student t-test was conducted for all such comparisons using open-source statistical programming language R http://r-project.org. Histograms of distributions were also plotted in the same package.

### Difference between bound and unbound pairs

For each protein chain in the RBP data set, a data set of monomeric proteins from PDB was scanned. Proteins with more than 90% similarity and coverage values were used as a pair of complexed and unbound monomers. Electric moments were then computed for both of them by the procedure described above. A total of 27 proteins were found to occur both in monomeric as well as RNA-complexed forms.

The difference between electric moments of a protein in its complexed and unbound forms is measured using Euclidean distance (ED) expression as follows:

(3) $$\text{ED}\left(\text{X}\right)\sqrt{\frac{1}{N}\sum {\left[X(bound)-X(free)\right]}^{2}}$$

Where X refers to dipole or quadrupole moment of the protein and summations is taken over all protein-pairs considered in a category (effectively a distance in 27-dimensional space).

### Neural network for prediction

A neural network-based predictor, similar to our earlier implementations (e.g. in [20]) was used to find a relationship between input vectors composed here of five descriptors based on charge, dipole moment and three eigenvalues of quadrupole moment and the functional property of protein chain e.g., binding or non-binding (control). To account for any cooperative and non-linear contribution of moments, a single hidden layer with 3 nodes has been used. To avoid over-assessment of performance, the neural network was trained in a jackknife style, by optimizing the predictor for all but one data in the training. Once the training is completed, prediction on the left-out protein is evaluated. After running through all binding and control proteins, overall prediction performance on the left-out proteins is evaluated. Since the neural network returns a real value between 0 and 1 for the target outputs 0 (non-binding) or 1 (binding), ROC data between specificity and sensitivity is calculated and converted to the area under the curve (AUC) values, which reflects performance over the entire range of cutoffs. Other measures of performance are as follows (T refers to true and F referes to false, whereas P is positive class and N is negative class):

(4) $$\begin{array}{l} \text{Precision}\left(\text{p}\right)=\text{TP}/\left(\text{TP}+\text{FP}\right)\\ \text{Recall}\left(\text{r}\right)=\text{TP}/\left(\text{TP}+\text{FN}\right)\\ \text{Accuracy}=\left(\text{TP}+\text{TN}\right)/\left(\text{TP}+\text{TN}+\text{FP}+\text{FN}\right)\\ \text{F}-\text{measure}=\text{2pr}/\left(\text{p}+\text{r}\right) \end{array}$$

F-measure is the geometric mean of precision and recall and can be computed by transforming real-valued outputs of neural network into binary class-label predictions at various cutoffs. Cutoffs at which F-measure has the highest value is used for reporting all class-wise performance measures, i.e. precision, recall, accuracy and F-measure.

## Results

### Statistics of electric moments

**Figure 1:**
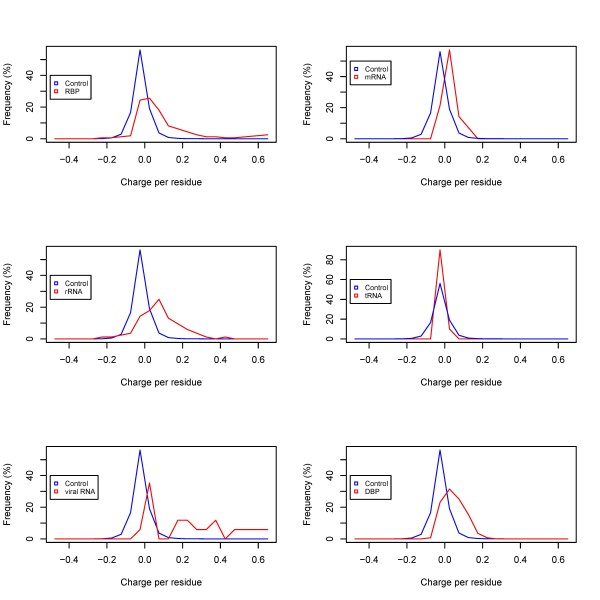
**Distribution of electric charges amongst RNA-binding proteins**. Abbreviations: First letter of each legend (q: charge, p: dipole moment, Q1: First eigen value of the quadrupole moment), followed by type of proteins considered (bind: all RNA-binding, dbp: DNA-binding, and proteins binding to tRNA (trna), mRNA (mrna) etc.)

**Figure 2:**
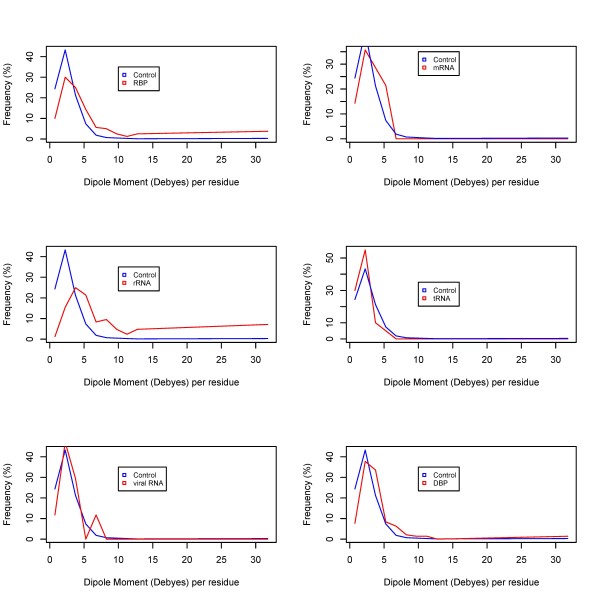
**Distribution of electric dipole moments amongst RBPs**.

**Figure 3:**
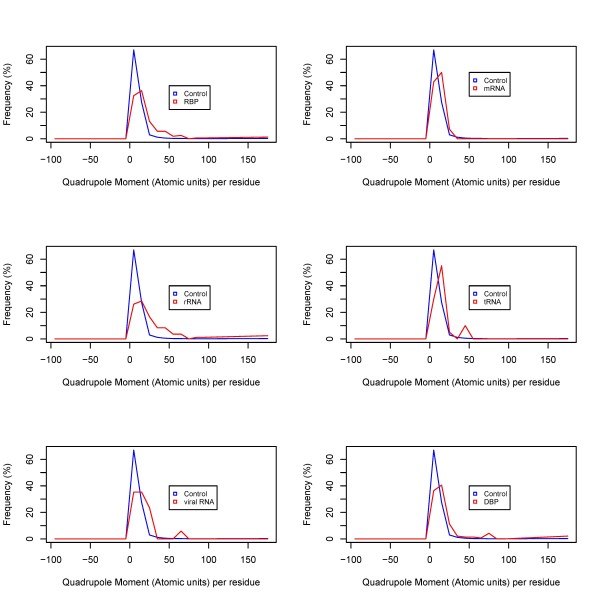
**Distribution of quadrupole moments amongst RBPs**.

**Figure 4:**
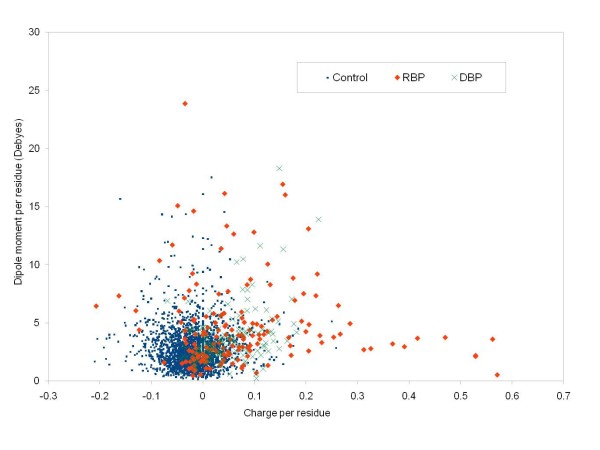
**Scatterplot of net charge versus dipole moment of RBP, DBP and control data sets**.

Figures 1, 2 and 3 show the frequency histograms of electric charge, dipole and quadrupole moments of RBPs compared with control as well as amongst various RBP classes. Figure 4 shows the detailed scatterplots of the most notable combinations. Table 1 shows the summary in terms of mean values. All the calculated electric moments of RBPs are provided in the Additional File 1. Observations from these results are summarized below.

**Table 1:** Mean and standard deviation values of electric moments in each class of RNA-binding protein (for a pair-wise comparison, see Table 2).

**Binding to**:	Mean (Charge)	Stdev	Mean (Dipole moment)	Stdev	Mean (Quadrupole moment)	Stdev
**RNA (any)**	0.075	0.129	4.613	3.681	20.156	20.455

**rRNA**	0.077	0.105	6.387	4.183	25.869	25.199

**tRNA**	-0.017	0.012	3.196	1.437	11.862	5.208

**viral RNA**	0.192	0.186	2.138	1.186	15.036	11.541

**mRNA**	0.025	0.037	3.728	2.499	16.616	14.455

**DNA**	0.048	0.057	2.991	1.817	20.226	29.061

**NB (Non-binding) (control)**	-0.020	0.043	2.664	1.748	9.972	9.294

#### Net charge

Average overall charge on control proteins is observed to be negative, which is consistent with our earlier control data sets used for analyzing DBPs [21]. We have observed that, similar to DBPs, RBPs also have an average overall positive charge (0.075 per residue) compared with negative (-0.020) values observed for control proteins. Statistical significance is also established by a *p*-value nearly zero (smaller than the precision limit of the software). However, the histogram in Figure 1 shows that there are a significant number (~40%) of RNA-binding proteins with negative or near zero net charge similar to control proteins. Further look at class-wide distributions shows that tRNA-binding proteins have almost no difference with control proteins in their distribution of charges (p-value ~0.24). On the other hand, mRNA-binding proteins have small difference compared to control data sets (p-values suggest that the difference is significant). However, the most significantly positively charged proteins are rRNA-binding and viral RNA-binding proteins (mean charge 0.077, 0.192 respectively), in which less than 20% proteins have negative or near zero net charge. This is statistically confirmed by the corresponding *p*-values (nearly zero) in comparison to control proteins (Table 2). When compared to DBPs (Table 1), RBPs are found to have even higher average charge than DBPs. However, looking at various RNA types, we observe that the higher charge on the average in RBP comes predominantly because of rRNA-binding proteins as they are the most abundant in the data set and have the highest positive charge. All other RBPs have significantly lower charge per residue than either the rRNA-binding proteins or DBPs.

**Table 2:** Pair-wise statistical significance (*p*-values) of difference in groups of RNA-binding proteins (for mean and standard deviation values in each group, see Table 1).

Group 1 binding to	Group 2 binding to	p-value (charge)	p-value (dipole moment)	p-value (quadrupole moment)
**RNA**	**NB**	<2.2E-016	4.19E-010	3.33E-009

**RNA**	**mRNA**	1.04E-003	6.08E-003	2.48E-004

**RNA**	**rRNA**	9.24E-001	1.30E-003	7.54E-002

**RNA**	**tRNA**	2.68E-015	1.69E-008	1.01E-001

**RNA**	**viral RNA**	2.09E-002	4.30E-003	3.69E-001

**RNA**	**DNA**	1.63E-002	1.40E-002	9.81E-001

**mRNA**	**rRNA**	1.41E-003	1.58E-006	1.66E-005

**mRNA**	**tRNA**	9.70E-004	3.26E-002	2.88E-001

**mRNA**	**DNA**	5.61E-002	2.37E-001	3.45E-003

**mRNA**	**viral RNA**	2.05E-003	7.30E-001	2.22E-001

**mRNA**	**NB**	5.09E-004	1.91E-001	2.01E-001

**rRNA**	**tRNA**	6.29E-012	1.86E-012	5.45E-003

**rRNA**	**viral RNA**	2.28E-002	1.66E-006	4.45E-002

**rRNA**	**DNA**	2.27E-002	5.46E-007	1.26E-001

**rRNA**	**NB**	1.05E-012	3.28E-012	1.30E-007

**tRNA**	**viral RNA**	2.73E-004	1.09E-001	7.19E-001

**tRNA**	**DNA**	<2.2E-016	2.21E-005	1.48E-001

**tRNA**	**NB**	2.40E-001	6.36E-002	6.50E-002

**DNA**	**viral RNA**	5.60E-003	1.44E-001	4.03E-001

**DNA**	**NB**	<2.2E-016	1.46E-006	4.53E-005

**viral RNA**	**NB**	2.34E-004	4.70E-001	7.66E-002

#### Dipole moment

We observe from Figure 2 that most RNA-binding proteins are distributed in the range of higher dipole moments. Overall, mean dipole moment for all RBPs is 4.6 units compared with 2.7 units for control proteins (with a highly significant *p*-value for the difference). Similar to the charge distribution, not all RBP types have higher dipole moments. However, interestingly, the classes with higher dipole moments are slightly different from those with higher positive charge. Although rRNA binding proteins continue to appear at the top of both lists, viral RNA-binding proteins are among the lowest dipole moments amongst all studied classes. On the lowest dipole moments side, tRNA-binding proteins, which have a charge distribution similar to control proteins (as observed above), also have a lower dipole moment (2.1), which is even lower than the control proteins. The highest dipole moment (average ~6.4 units) for rRNA-binding proteins suggests a predominant role for electrostatic interactions in the ribosomal complexes.

#### Quadrupole moment

Histograms of quadrupole moments show a relatively more subtle role in RBPs. Average values of quadrupole moments in RBPs and DBPs are very similar, but again this comes mainly from rRNA-binding proteins, as all other types of RBPs have lower quadrupole moments than rRNA-binding or DNA-binding proteins.

#### Collective role of moments

It may be possible that proteins which are not well classified from each other in terms of a single electric moment or charge may be better classified in their combined values. Role of a pair of moments in such recognition can be observed from their scatterplots. Figure 4 shows that a number of RBPs have a higher charge but no significant dipole moment (no DBPs are observed in this category). Most of these proteins are binding to viral RNA. Similarly, some RBPs have a higher dipole moment but no positive charge. Again, there are fewer DBPs in this category. There is a tendency for DBPs that an increased charge also leads to increased dipole moment, but not always the case for RBPs. In other words, positive charges in DBPs are likely to be more localized compared to RBPs, increasing the dipole moments only in the former proteins. A similar difference exists in terms of quadrupole moments of these two types of nucleic acid binding proteins (data not shown).

### Neural network based prediction

We observed above that the electric moments of all RBPs differ from control proteins as well as among their subclasses. However, as shown in the scatterplots and Table 1, individual group of proteins may not simply be identified by a single descriptor. For example, rRNA-binding proteins have higher values for all three moments, whereas viral RNA-binding proteins are better characterized by only the total charge. To determine the cumulative contributions of these features in protein-RNA recognition, we designed a neural network and trained it to take advantage of all of these features. Neural network performance in distinguishing any two types of proteins is measured by the area under the ROC curve and results are shown in Table 3. The results indicate that RBPs can be distinguished from control proteins at nearly 78% accuracy. However, rRNA-binding proteins could be determined at even higher accuracy (~79%). This is understandable as we show above that all three discussed properties in rRNA-binding proteins are significantly higher than any other category discussed, including DBPs. Some groups of proteins such as tRNA-binding and mRNA-binding proteins could not be distinguished from control at all, showing over-fitting for training data and almost no generalization value in the trained neural network. Also, DBPs and RBPs, despite subtle differences in their distributions, show limited difference when all factors are taken into account suggesting that the diversity in their moments is more than the amount of data and that many more features will be needed for such a fine-tuning of classification. Results of this classification are consistent with more detailed prediction obtained by up to 40 descriptors [12]. Authors in that study report nearly 81% AUC for identifying RBPs from control data using 10 electrostatic features. Our results, based on a much larger data and just three features, reached a performance of 78% AUC, which is comparable in performance, keeping in view that we do not apply the method to a specific patch but use the whole protein, and thereby show that the method can be used in a more general framework without much loss of performance. We have also developed pair-wise prediction models for various protein classes rather than just the comparison with control data sets, which has not been attempted earlier. With regards to the discrimination between DBPs and RBPs, we reach the same conclusion as [12], i.e. these two classes cannot be distinguished from each other with much confidence.

**Table 3:** Neural network performance to discriminate between proteins binding to different types of RNA based on charge, dipole and quadrupole moments*.

Positive class binding to	Negative class binding to	Number of proteins in + ve class	Number of proteins in -ve class	AUC	F1	Precision	Recall	Accuracy
**RNA**	**NB**	160	2441	0.78	0.37	0.31	0.45	0.91

**rRNA**	**NB**	84	2441	0.79	0.26	0.23	0.30	0.94

**tRNA**	**NB**	20	2441	0.42	0.02	0.01	1.00	0.03

**vRNA**	**NB**	17	2441	0.75	0.24	0.24	0.24	0.99

**mRNA**	**NB**	13	2441	0.10	0.01	0.01	1.00	0.02

**tRNA**	**rRNA**	20	84	0.70	0.45	0.32	0.75	0.64

**mRNA**	**rRNA**	13	84	0.56	0.30	0.18	1.00	0.37

**vRNA**	**rRNA**	17	84	0.44	0.32	0.19	1.00	0.28

**mRNA**	**tRNA**	13	2441	0.07	0.57	0.39	1.00	0.39

**mRNA**	**vRNA**	13	2441	0.02	0.60	0.43	1.00	0.43

**tRNA**	**vRNA**	20	17	0.19	0.63	0.46	1.00	0.46

**DNA**	**NB**	143	2441	0.72	0.22	0.20	0.26	0.90

**RNA**	**DNA**	160	143	0.58	0.69	0.53	1.00	0.53

**rRNA**	**DNA**	84	143	0.74	0.64	0.52	0.83	0.65

**tRNA**	**DNA**	20	143	0.33	0.24	0.13	1.00	0.20

**mRNA**	**DNA**	13	143	0.07	0.16	0.09	1.00	0.14

* AUC is area under the ROC curve, F-measure (F1) is the highest geometric mean of precision and recall and accuracy is number of correct predictions relative to all predictions at peak F-measure. In all cases, neural network with three units in the hidden layer was used for training in a leave-one-out procedure and the training was performed for a fixed number of epochs without using information from left-out protein.

### Bound versus unbound states

**Figure 5:**
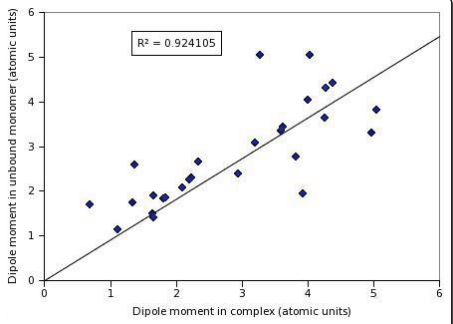
**Dipole moments of RNA-binding proteins in complexed structure compared with their independently solved monomeric form**.

**Figure 6:**
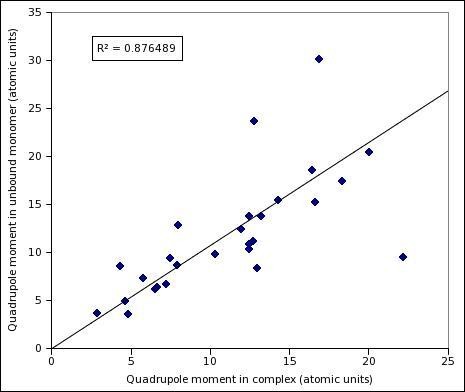
**Quadrupole moments of RNA-binding proteins in complexed structure compared with their independently solved monomeric form**.

**Figure 7:**
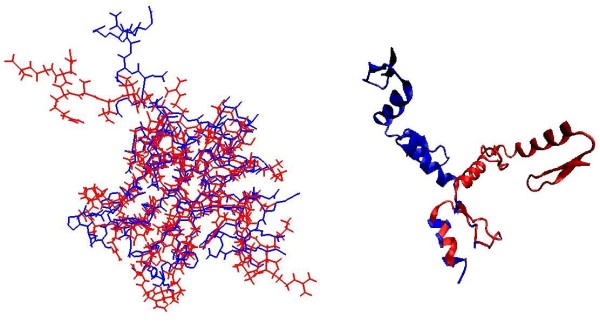
**Superimposed structures of pairs of RBPs in RNA-complexed structure and their unbound monomeric forms**. Figure on the left shows complexed and unbound monomeric pairs of 30S ribosomal protein S16 (Complex PDB ID 1hnw_P in red, unbound PDB ID 1emw_A in blue) and on the right a pair of Zinc finger structures in complex (1un6_B, blue) and unbound forms (2j7j_A, red) have been shown. Dipole and quadrupole moment values for Ribosomal protein S16 remain almost unchanged despite undergoing conformational changes (Table 5), whereas zinc finger pairs show a significant difference in the two variations. However, this protein (zinc finger) is a rare example of very large conformational changes in RBPs and in the compared pairs in Table 4, is the only exception to all other pairs, where complex and unbound structures have similar values of moments. This exception was further analyzed to reveal that the moments in the individual domains remain largely unchanged.

Most solved as well as modeled protein structures come from monomeric forms and formation of a full complex from isolated RBPs may lead to structural changes, which may make the predictions performed on complex-derived structures questionable. Prima facie, it appears that the calculations of moments using low-resolution structure information (C_α _atoms only) will be more robust than existing methods utilizing side-chain coordinates coming from complexes at high resolution. To assess the validity of this intuitive argument, we compiled a list of structure-pairs of RBPs: one member of each pair came from the complex and the other from a monomer with no other protein or RNA (this may involve conformational changes by protein-protein or protein-RNA interactions). Detailed procedure for selecting pairs is described in Methods. Table 4 gives detailed comparison between the dipole moment and quadrupole moment of these structure pairs (charge is obviously identical in the two cases). Figure 5 and 6 show the scatterplots of dipole and quadrupole moments observed in monomeric and complexed structures. It is clear that correlation coefficients are close to 1 for both dipole and quadrupole moment values. Clearly, the calculation of moments from C_α _atoms only makes the procedures far more robust than any other electrostatic property calculated from full atomic coordinates. Figure 7 shows a typical pair of structures as well as an exceptional case with very large conformational change in complex formation. Figure on the left shows complexed and unbound monomeric pairs of 30S ribosomal protein S16 (Complex PDB ID 1hnw_P, unbound PDB ID 1emw_A), and on the right a pair of Zinc finger structures in complex (1un6_B) and unbound forms (2j7j_A) are shown. Dipole and quadrupole moment values for Ribosomal protein S16 remain almost unchanged despite undergoing conformational changes (Table 4), whereas zinc finger pairs show a significant difference in the two variations. However, this protein has been shown to have two modes of binding via changes in domain orientations. When the moments of each domain were calculated separately, bound and the unbound conformations were found to have very similar moments, confirming that the dipole and electric moment values are fairly robust against small conformational changes induced by complex formation (see Table 5).

**Table 4:** Electric moments of RNA-binding proteins as pairs of RNA-complexed and monomeric structures*.

	Detailed protein-wise comparison				ED
**Protein name**	**P**		**Q**		

	**Complex.**	**Monomer.**	**Complex.**	**Monomer.**	

30S Ribosomal protein S15 (1fjgO, 2fkxA, 100%)	4.37	4.44	5.77	7.36	rRNA binding: ED(P) = 0.7 ED(Q) = 1.6
	
30S Ribosomal protein S6 (1fjgF, 1louA, 99%)	2.34	2.66	7.90	8.74	
	
30S Ribosomal protein S7 (1fjgG, 1rssA, 100%)	4.95	3.30	22.12	9.56	
	
30S Ribosomal protein S19 (1ibmS, 1qkfA, 100%)	5.04	3.83	12.96	8.43	
	
30S Ribosomal protein S16 (1hnwP, 1emwA, 100%)	4.27	4.31	7.22	6.76	
	
Ribosomal protein L11 (1hc8A, 2f0wA, 100%)	1.79	1.84	6.64	6.41	
	
Ribosomal protein L25 (1d6kA, 1b75A, 100%)	3.59	3.35	10.26	9.90	
	
60S Ribosomal protein L30 (1cn8A, 1cn7A, 100%)	2.94	2.41	4.61	4.97	

Glutaminyl-tRNA synthetase (1euyA, 1nylA, 98%)	1.10	1.15	14.24	15.46	tRNA binding: ED(P) = 0.6 ED(Q) = 1.2
	
Queuine tRNA-ribosyltransferase (1q2rA, 1r5yA, 100%)	2.19	2.26	2.83	3.75	
	
Glutamyl-tRNA synthetase (1g59A, 1j09A, 99%)	1.64	1.50	13.21	13.77	
	
Aspartyl tRNA-synthetase (1asyA, 1eovA, 100%)	4.03	5.06	7.48	9.39	
	
Elongation factor TU (1b23P, 2c78A, 98%)	3.62	3.44	12.65	11.21	
	
Arginyl tRNA synthetase (1f7uA, 1bs2A, 100%)	2.22	2.31	12.44	13.83	
	
Small protein B (1p6vA, 1k8hA, 98%)	0.69	1.71	16.40	18.62	
	
Pseudouridine synthase B (1k8wA, 1r3fA, 100%)	1.65	1.90	18.31	17.41	
	
Tyrosyl tRNA synthetase (1j1uA, 2ag6A, 96%)	1.34	1.75	16.60	15.22	

Bactereophage coat protein MS2 (1aq3A, 1mscA, 98%)	2.08	2.08	4.77	3.65	Viral RNA binding: ED(P) = 0.7 ED(Q) = 1.9
	
Minor core protein lambda 3 (1n1hA, 1mukA, 100%)	1.65	1.42	11.95	12.44	
	
RNA polymerase HC-J4 (1nb7A, 1gx5A, 96%)	3.81	2.77	12.44	10.34	
	
HIV-I nucleocapsid protein (1a1tA, 1mfsA, 100%)	4.24	3.65	12.74	23.69	

NHP2-like protein 1 (1e7kA, 2jnbA, 100%)	3.27	5.06	4.28	8.61	Others: ED(P) = 0.9 ED(Q) = 2.0
	
Splicosomal U1A protein (1audA, 1fhtA, 98%)	1.36	2.60	7.93	12.88	
	
Pumilo homology domain (1m8yB, 1m8zA, 100%)	4.00	4.05	20.01	20.46	
	
Rho transcription termination factor (2a8vA, 1a62A)	1.84	1.86	12.42	10.87	
	
Transcription factor IIIA (**) (1un6B, 2j7jA, 100%)	3.92	1.94	16.83	30.18	
	
VP39 protein (1av6A, 4dcgA, 98%)	3.18	3.09	6.53	6.25	

*P and Q stands for dipole moment and quadrupole moment (first eigen value) respectively. Euclidean distance (ED) here refers to the root mean squared difference between bound and unbound moments for the given pair. (**) This protein is reported to have two modes of interaction and shows very large conformational change via domain movement (rigid body RMSD is 9.2Å). However, domain-wise comparison shows almost no change in moments (see Table 5).

**Table 5:** Electric moments of three domains in Zinc finger, which undergoes very large conformational change.

Domain	Dipole moment (bound; 1un6)	Dipole moment (unbound; 2j7j)	Quadrupole moment (bound; 1un6)	Quardrupole moment (unbound; 2j7j)
Domain I (1-28)	5.5	5.4	5.1	5.3

DomainII (29-57)	6.0	6.0	7.6	6.2

Domain III (58-87)	3.3	3.6	4.3	4.8

### Evolutionary conservation

We also examined if the electric moments, calculated above, remain conserved among homologous or similar RNA-binding proteins. To evaluate this, we returned to the overall data of RNA-binding proteins (before removing redundancy), obtained during the process of selecting representative non-redundant set of 160 proteins. Out of 160 clusters obtained above 57 contained at least 10 members each and we plotted the noise to signal (N/S) ratio (standard deviation within each cluster relative to the mean value for each of the three electric moments). By looking at the N/S ratio (data not shown), we find that the charge and dipole moments are highly conserved within the family: the N/S ratio in net charge is less than 2% for most of the proteins, whereas for the dipole moment, most data is within 5% range of mean. In the case of quadrupole moments, the variation is slightly more, suggesting that quadrupole moment may not be as strictly conserved due to the flexibility of structure. However, even quadrupole moments show a fairly conserved distribution and whatever variations in this features are caused by evolutionary or structural variations are relatively small and are not likely to affect the predictability of such proteins from structure.

### Protonation state of Histidine residues

Although some methods to predict protonation state of His residues are available, we adopted a more straightforward approach, which does not require knowledge of side chain atomic coordinates i.e. by treating all His residues as neutral. To estimate how far this will affect the conclusions of this study, we created the other extreme case i.e., when all His are treated to have a positive charge. We find that the correlation between the charge, dipole moments and quadrupole moments in these two extreme cases are 0.99, 0.98 and 0.96 respectively (we take the best correlated pair from the three values of quadrupole moment eigen values to quote this value). Together with a good prediction performance obtained by our method of assigning charges, this justifies ignoring the protonation of His residues.

### A practical example

**Figure 8:**
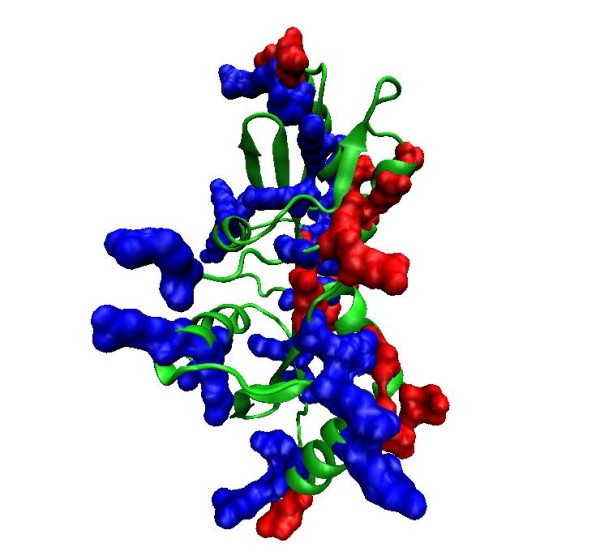
**Distribution of positively charged (Lys and Arg) residues (blue surface filled) and negatively charged (Asp and Glu) residues (red surface filled) in HI1333, a hypothetical protein from Haemophilus influenzae **(PDB ID 1JO0)** from the protein data bank**. Protein has a significantly high dipole moment and its RNA-binding region seems to be clearly separated from the negatively charged region by a vertical plane.

The analysis presented above shows that electric moments are a useful indicator of proteins to annotate them as RNA-binding. To illustrate the use of this study, we computed the electric moments of a hypothetical protein from PDB (PDB ID 1JO0) i.e. HI1333, which is a hypothetical protein from Haemophilus influenzae and it has been marked as candidate of being an RNA-binding protein [22]. We computed the electric moments of this protein by our method and found that the net charge and dipole moment of this protein are 0.026 and 4.22 Debyes respectively. Both these values are high and support the view that this could be an RNA-binding protein. To examine its charge distribution further, we plotted the distribution of Arg and Lys (positively charged) and Glu and Asp (negatively charged) residues shown in blue and red respectively in Figure 8. We see that there is a clear separation of positive and negatively charged regions along the horizontal axis of the figure; positively charged residues are protruded to the left, giving rise to a high dipole moment. In addition to the current supporting view of its annotation as RNA-binding protein, this analysis suggests a possible mechanism of interaction i.e., through a dipole moment, which possibly steers it into the negatively charged scaffold of target RNA molecule.

## Discussion

The main results presented above show that, similar to DNA-binding proteins, RNA-binding proteins also show a bias in the distribution of their basic electrostatic features. However, the dipole and quadrupole moments for proteins which bind to ribosomal RNA stand out in comparison with all other classes, suggesting that the main driving force for the formation and functioning of ribosomal assembly has strong electrostatic character revealed not only by their overall charge but orientations and spherical asymmetry contained in higher values of moments. Interaction of proteins with the transcribed RNA is highly order-specific as some proteins bind only after some others are already bound to the partially transcribed RNA [23]. Exact order of presenting proteins to the RNA may require quick recognition, and dipole-dipole and quadrupole interactions may facilitate this process by their long-range steering effect. Some high-resolution studies on specific electrostatic interactions such as the one formed by non-bridging phosphate oxygen have also been reported [24]. Thus, it is speculated that the requirement of orderly assembly and stabilizing electrostatic interactions, which are specific to rRNA-protein interactions, are reflected in higher electric moments in proteins interacting with them. Such an order of events is not essential in other types of RNA molecules as they do not have similar process of assembly (for example, tRNA interactions with proteins have been reported to have a clearly distinct mechanism, although they are also guided by electrostatic forces [25]).

Although studies specifically trying to optimize prediction performance using structure-based bulk electrostatic properties have been reported, they largely focus on charged patches and their geometry in RBPs. We have on the other hand analyzed only three electrostatic properties in more details and used the whole protein as the input for prediction model. This allowed us to examine how the charge distribution may characterize mode of action for these proteins. For example, predominant role of charge and dipole moment in ribosomal proteins stands out as explained above. Another group of RBPs that emerges distinct from this study is viral RNA-binding proteins, which have high amount of charge but not the dipole moment, making this group of proteins distinct from others in terms of a remarkably symmetric distribution of (unbalanced) charge over their surface. Thus, in utilizing charge and its asymmetric distribution on surface, rRNA-binding proteins form an extreme group, whereas other proteins utilize one or more of the three measures considered here. Based on this, the prediction performance of a model using just these properties remains comparable with more detailed methods. Furthermore, additional insight into the mechanism of action of RNA-protein recognition in various functional groups is obtained.

Another key observation in this work is that the change in electric moments due to complex formation is not large, unless it is accompanied by very large conformational changes as in the case of domain movement and multiple proteins with multiple binding modes. However, even in these cases, the constituent domains likely maintain their overall multi-polar electrostatic properties. It may be noted that the pair-wise data set of bound/unbound proteins in this work is somewhat biased as proteins whose structures change considerably are more likely to be reported. This is confirmed by measuring their RMSD after superimposing the structures (we find that the average RMSD of all pairs is 2.1Å, which is quite large; data not shown). Thus, despite these large conformational changes, characteristic electric moments are largely preserved, probably helping in long-range interactions resulting in appropriate energy landscape for recognition by steering.

We observe that the three electric moments are fairly conserved in evolution, and even sequence similarity being as low as 25%, RNA-binding proteins within the same cluster seem to have very similar electric moments, suggesting that the three properties may be universally employed for protein-RNA recognition.

Finally, this method has been rigorously cross-validated on known protein structures of RNA-binding proteins. However, the most useful application of the method would be to annotate proteins from their modeled structures. Unfortunately, a readily available public data of modeled structures with RNA-binding annotations was not available at the time of this study. Thus, all performance measures presented here correspond to real structures (although with very lenient requirements of resolution). Benchmarking performance on high throughput modeled structures remains an area for further investigation.

## Conclusions

RNA-binding proteins have distinct patterns of net charge, dipole and quadrupole moments, which can be utilized to rapidly identify them and to some degree determine their structure class. This information is present even at a low-resolution level, as moments calculated from only main-chain coordinates can be utilized for prediction. This method is also robust against conformational changes, as well as evolutionary variations in protein structures.

## Authors' contributions

This project was jointly conceived by both authors (SA and AS), as part of their ongoing collaborations. Detailed experimental design and implementation were carried out by SA. Manuscript preparation and analysis of results were carried out by SA in consultation with and suggestions from AS. Both authors read and approved the manuscript.

## Supplementary Material

Additional file 1 **Electric Moments of RNA-binding proteins**. Charge (q), dipole moment (p) and quadrupole moments (three eigen values, Q1, Q2, Q3) of RNA-binding proteins. DNA-binding proteins and control proteins are also included. Click here for file
